# Diagnostic efficacy of noninvasive liver fibrosis indexes in predicting portal hypertension in patients with cirrhosis

**DOI:** 10.1371/journal.pone.0182969

**Published:** 2017-08-18

**Authors:** Le Wang, Yuemin Feng, Xiaowen Ma, Guangchuan Wang, Hao Wu, Xiaoyu Xie, Chunqing Zhang, Qiang Zhu

**Affiliations:** 1 Department of Gastroenterology, Shandong Provincial Hospital Affiliated with Shandong University, Jinan, Shandong, China; 2 Shandong Provincial Engineering and Technological Research Center for Liver Disease Prevention and Control, Jinan, Shandong Province, China; Kaohsiung Medical University Chung Ho Memorial Hospital, TAIWAN

## Abstract

**Background:**

Recent data suggest that noninvasive liver fibrosis indexes could be useful for predicting esophageal varices (EV) in cirrhotic patients. However, thus far, the diagnostic efficacy of these indexes in predicting portal hypertension (PH) in cirrhotic patients has been poorly evaluated.

**Aims:**

To evaluate the diagnostic efficacy of noninvasive liver fibrosis indexes in the diagnosis of PH.

**Methods:**

A total of 238 cirrhotic patients underwent hepatic venous pressure gradient (HVPG) evaluation and relevant serum tests to analyze the variables associated with PH grade. Then, the diagnostic performances of seven fibrosis indexes, the aspartate aminotransferase (AST)-to-alanine aminotransferase (ALT) ratio (AAR), AST-to-platelet (PLT) ratio index (APRI), fibrosis index (FI), FIB-4, Forns index, King’s score and the Lok index, were evaluated to determine their efficacy in predicting clinically significant PH (CSPH) and severe PH (SPH). In addition, the performances of these fibrosis indexes in different subgroups were investigated.

**Results:**

The results of a multivariate analysis of serum markers showed that AST values, platelet (PLT) count and albumin (ALB) were associated with PH grade. Among the seven—fibrosis indexes, King’s score, APRI and the Lok index showed modest diagnostic accuracy in predicting CSPH and SPH, as indicated by AUC of 0.755 and 0.742, 0.740 and 0.742, and 0.722 and 0.717, respectively. In addition, combination of King’s score (cutoff 23.47) and Lok index (cutoff 1.30) predicted presence of CSPH, with the highest PPV (95.38%) and +LR (5.49). A subgroup analysis indicated that the noninvasive screening model may be more applicable to patients with cirrhosis of viral etiology.

**Conclusions:**

Serum liver fibrosis indexes exhibited modest diagnostic accuracy for PH in cirrhotic patients. These indexes may not be able to replace HVPG measurements for the diagnosis of PH but may be used as a first-line screening method for CSPH in liver cirrhosis patients.

## Introduction

Liver cirrhosis is the most common cause of portal hypertension (PH), which leads to severe complications, such as esophageal varices (EV), ascites and decompensation[1,2]. PH and its complications account for the majority of morbidity and mortality in patients with chronic liver diseases. Studies have indicated that the early diagnosis of PH is necessary for timely treatment[3]. Currently, measurement of the hepatic venous pressure gradient (HVPG) is considered the gold standard for PH assessment and is the most important predictor of complications arising from PH in cirrhotic patients[4]. However, HVPG measurement is an invasive procedure that requires technical expertise, which is available in only a few centers. Therefore, the need for simple and convenient noninvasive alternatives has become urgent.

Recently, many laboratory, clinical and ultrasonographic variables have been evaluated as noninvasive alternatives to HVPG measurement[5–7]. However, none of them could be recommended in everyday clinical practice due to inadequate accuracy or poor validation. Among the noninvasive alternative methods, serum markers are simple and easily evaluated in the clinic. Recently, various serum fibrosis markers have been explored as predictors of EV[8–10]. However, the diagnostic efficacy of these liver fibrosis indexes in predicting PH in patients with cirrhosis has been poorly evaluated thus far. It is generally accepted that the pathophysiology of PH includes increases in intrahepatic vascular resistance (IVR) and portal blood inflow, with the former representing the primary factor[11,12]. Therefore, based on the concept that PH is mainly attributed to increased vascular resistance caused by hepatic fibrosis, we speculated that serum liver fibrosis indexes may also be used as surrogate markers of PH.

In this study, we evaluated serum markers that are easily measured in clinical practice and compared the diagnostic performance of a series of recently proposed noninvasive fibrosis indexes as a new predictor of PH in patients with cirrhosis and an alternative to HVPG measurement. We also examined whether combining the markers might increase their diagnostic accuracy for predicting PH and help stratify the grade of PH in cirrhotic patients as a noninvasive first-line screening method.

## Materials and methods

### Patients

All eligible patients with a diagnosis of liver cirrhosis who were consecutively admitted between January 2012 and June 2015 were enrolled in this retrospective study based on the following criteria. The inclusion criteria were as follows: patients with liver cirrhosis who underwent both laboratory tests and HVPG measurement. The diagnosis of liver cirrhosis was based on histology, clinical or imaging data, and at least met one of the following criteria: (1) biopsy-proven stage 4 fibrosis; (2) clinical liver decompensation including ascites, variceal hemorrhage, and hepatic encephalopathy; and (3) imaging signs showing a nodular and shrunken liver [13,14].

Patients with the following criteria were excluded: lack of HVPG measurement or relevant laboratory data; splenectomy; pregnancy; malignant tumors; liver transplantation; and serious disease in other organ systems. All information regarding patient demographics, laboratory data, etiology of cirrhosis, Child-Pugh classification and HVPG measurement was obtained from the electronic medical records of Shandong Provincial Hospital Affiliated with Shandong University, and the study began on June 2, 2016. The authors had no access to information that could identify individual participants during or after data collection. All procedures were approved by the Ethics Committee of Shandong Provincial Hospital Affiliated with Shandong University. In addition, all methods were performed in accordance with the relevant guidelines and regulations.

### Measurement of HVPG

HVPG measurements were performed for each patient by one experienced operator according to the international standard[15,16]. Free hepatic venous pressure (FHVP) was measured by placing a balloon catheter in the right hepatic vein through right jugular vein puncture. Then, the operator inflated the balloon catheter in the right hepatic vein to measure the wedged hepatic venous pressure (WHVP). Finally, the HVPG value was determined by subtracting the FHVP from the WHVP. Measurements were performed at least three times and averaged. According to the consensus, clinically significant PH (CSPH) was diagnosed as HVPG ≥ 10 mmHg, whereas severe PH (SPH) was diagnosed as HVPG ≥ 12 mmHg. HVPG < 10 mmHg was defined as PH grade 1; 10 ≤ HVPG < 12 mmHg was defined as PH grade 2; and HVPG ≥ 12 mmHg was defined as PH grade 3.

### Noninvasive liver fibrosis indexes

The following serum markers were evaluated in all patients using published formulas: aspartate aminotransferase (AST)-to-alanine aminotransferase (ALT) ratio (AAR), AST-to-platelet (PLT) ratio index (APRI), fibrosis index (FI), FIB-4, Forns index, King’s score, and the Lok index, as shown below[17–23].

AAR=AST÷ALTAPRI=[(AST÷upperlimitofnormal(ULN))×100]÷PLT

$$FI=8-0.01\times PLT-ALB\;FIB-4=\left(age\times AST\right)÷PLT\times \sqrt{ALT}\;\mathrm{FIB}\text{-}4=(\mathrm{age}\times \mathrm{AST})÷\mathrm{PLT}\times \surd \mathrm{ALT}$$

King’sscore=age×AST×internationalstandardratio(INR)÷PLT

$$\begin{array}{l} Forns\;index=7.811-3.131\times \ln\left[PLT\right]+0.781\times \ln\left[gamma-glutamyl\;transferase\left(GGT\right)\right]+\\ 3.467\times \ln\left[age\right]-0.014\times \left[cholesterol\right] \end{array}$$

Lokindex=−5.56−0.0089×PLT+1.26×(AST÷ALT)+5.27×INR

### Statistical analysis

Continuous variables are expressed as the means ± standard deviation (SD), and categorical data are expressed as numbers (percentages). The nonparametric Mann–Whitney test was used to analyze differences between groups, and the χ2 test or Fisher’s exact test was applied for the comparison of categorical data. With the aim of identifying variables independently associated with HVPG, a multivariate analysis was performed using the ordinal logistic-regression procedure on variables that were significantly different in the univariate analysis. A receiver operating characteristic (ROC) analysis was used to assess the diagnostic performance of each noninvasive test in detecting CSPH and SPH, and each area under the ROC curve (AUC) was calculated[24]. The optimal cutoff values were chosen using the Youden index. The diagnostic value of each noninvasive index was calculated based on sensitivity, specificity, accuracy, positive predictive value (PPV), negative predictive value (NPV), positive likelihood ratio (+LR), negative likelihood ratio (-LR) and 95% confidence intervals (CIs). ROC curves were compared as described by Hanley et al[25].

All statistical analyses were performed using SPSS v22.0 software (SPSS Inc, Chicago, IL, USA) or MedCalc Statistical Software version 15.2.2 (MedCalc Software bvba, Ostend, Belgium). For all analyses, p-values were two-tailed, and p-values < 0.05 were considered statistically significant.

## Results

### Baseline patient characteristics

A total of 238 patients, including 161 males and 77 females, were eligible for inclusion in the study (S1 Appendix). The main demographic, laboratory and clinical characteristics of the patients and the features of the CSPH and SPH subgroups are listed in Table 1. Among the included patients, 188 (78.99%) had CSPH, and 163 (68.49%) had SPH.

**Table 1:** Baseline characteristics of patients with cirrhosis.

Variables	Total (n = 238)	Patents without CSPH (n = 50)	Patents with CSPH (n = 188)	p	Patents without SPH (n = 75)	Patents with SPH (n = 163)	p
**Age (y) mean** ± **SD**	52.55 ± 10.44	53.74 ± 10.23	52.24 ± 10.50	0.463	52.35 ± 11.27	52.64 ± 10.07	0.899
**Sex (M/F), n**	161/77	31/19	130/58	0.338	50/25	111/52	0.827
**Cause of cirrhosis, n (%)**				0.003			0.016
**Viral cause**							
**Hepatitis B virus**	122(51.26%)	19(38.00%)	103(54.79%)		33(44.00%)	89(54.60%)	
**Hepatitis C virus**	13(5.46%)	0(0.00%)	13(6.91%)		1(1.33%)	12(7.36%)	
**Non-viral cause**							
**Alcohol**	20(8.40%)	3(6.00%)	17(9.04%)		3(4.00%)	17(10.43%)	
**Primary biliary cirrhosis**	7(2.94%)	1(2.00%)	6(3.19%)		1(1.33%)	6(3.68%)	
**Autoimmune**	10(4.20%)	4(8.00%)	6(3.19%)		4(5.33%)	6(3.68%)	
**Cryptogenic**	66(27.73%)	23(46.00%)	43(22.87%)		33(44.00%)	33(20.25%)	
**Child-Pugh classification, n (%)**				<0.001			<0.001
**A**	110(46.22%)	39(72.00%)	71(37.77%)		55(73.33%)	55 (33.74%)	
**B**	104(43.70%)	11(22.00%)	93(49.47%)		20(26.67%)	83(50.92%)	
**C**	24(10.08%)	0(0.00%)	24(12.77%)		0(0.00%)	25(15.33%)	
**Ascites, n (%)**				<0.001			<0.001
**No**	111(46.64%)	34 (68.00%)	77(40.96%)		50(66.67%)	59(36.20%)	
**Mild**	67(28.15%)	15(30.00%)	52(27.66%)		23(30.67%)	44(26.99%)	
**Moderate-severe**	60(25.21%)	1(2.00%)	59(31.38%)		2(2.67%)	60(36.81%)	
**AST(IU/L), mean ± SD**	47.21 ± 34.19	32.38 ± 13.91	51.15 ± 36.83	<0.001	34.77 ± 20.00	52.93 ± 37.72	<0.001
**ALT(IU/L), mean ± SD**	39.19 ± 40.47	24.86 ± 10.79	43.01 ± 44.45	<0.001	29.12 ± 21.44	43.83 ± 46.02	0.002
**GGT(IU/L), mean ± SD**	61.04 ± 70.80	56.55 ± 82.76	62.23 ± 67.47	0.094	56.98 ± 82.78	62.91 ± 64.75	0.024
**Albumin (g/dL), mean ± SD**	33.29 ± 6.97	36.10 ± 7.23	32.55 ± 6.73	0.001	35.77 ± 7.55	32.15 ± 6.4	<0.001
**Total bilirubin (mg/dL), mean ± SD**	31.53 ± 40.06	23.06 ± 10.74	33.78 ± 46.78	0.089	22.58 ± 10.32	35.64 ± 49.85	0.007
**Cholesterol(mmol/L)**	3.81 ± 1.25	3.82 ± 0.99	3.81 ± 1.31	0.667	3.95 ± 1.23	3.78 ± 1.25	0.253
**PLT count (109/L), mean ± SD**	99.50 ± 70.08	130.64 ± 90.30	91.21 ± 60.92	0.002	123.65 ± 93.27	88.38 ± 53.01	0.004
**Prothrombin time (%), mean ± SD**	14.83 ± 1.93	13.80 ± 1.22	15.11 ± 1.99	<0.001	14.15 ± 1.67	15.15 ± 1.97	<0.001
**INR, mean ± SD**	1.24 ± 0.17	1.15 ± 0.11	1.27 ± 0.17	<0.001	1.17 ± 0.12	1.27 ± 0.18	<0.001

NOTE. Data are expressed as the means ± standard deviation or as percentages.

Abbreviations: SD, standard deviation; M, male; F, female; AST, aspartate aminotransferase; ALT, alanine aminotransferase; GGT, gamma-glutamyl transferase; PLT, platelet; INR, international standard ratio; and HVPG, hepatic venous pressure gradient.

### Factors associated with HVPG grade by univariate analysis

To analyze variables associated with the presence of PH, we evaluated markers easily detectable in clinical practice. The variables associated with the presence of CSPH, SPH and HVPG grade were analyzed using univariate analysis. Among all the variables presented in Table 1, factors associated with the presence of CSPH were etiology, Child-Pugh score, ascites, AST values, ALT values, ALB, PLT count, prothrombin time and INR. Moreover, factors associated with SPH, such as etiology, Child-Pugh score, ascites, AST values, ALT values, ALB, GGT, total bilirubin, PLT count, prothrombin time and INR, were significantly associated with the presence of SPH.

Finally, factors associated with HVPG grade were etiology, Child-Pugh score, ascites, AST values, ALT values, ALB, PLT count, prothrombin time and INR.

### Factors associated with HVPG grade by multivariate analysis

To ensure that all the evaluated factors associated with HVPG grade were simple and easily detectable serum markers, variables such as etiology, Child-Pugh score and ascites were omitted from the multivariate analysis by the ordinal logistic-regression procedure. Thus, the variables independently associated with HVPG grade were as follows: AST value (OR 1.033, 1.031–1.034 95% CI, p = 0.005), PLT count (OR 0.993, 0.990–0.995 95% CI, p = 0.002) and ALB (OR 0.943, 0.940–0.947 95% CI, p = 0.026), which were used to construct the HVPG prediction model. However, the performance of the model in predicting CSPH and SPH was not satisfactory, with an AUC of 0.780 (0.722–0.831 95% CI) and 0.769 (0.711–0.821 95% CI); sensitivity of 68.62% and 69.33%; specificity of 80% and 74.67%; PPV of 92.81% and 85.61%; NPV of 40.41% and 52.83%; +LR of 3.43 and 2.72; and -LR of 0.39 and 0.41, respectively.

### Performance of serum liver fibrosis indexes in the detection of CSPH

**Fig 1:**
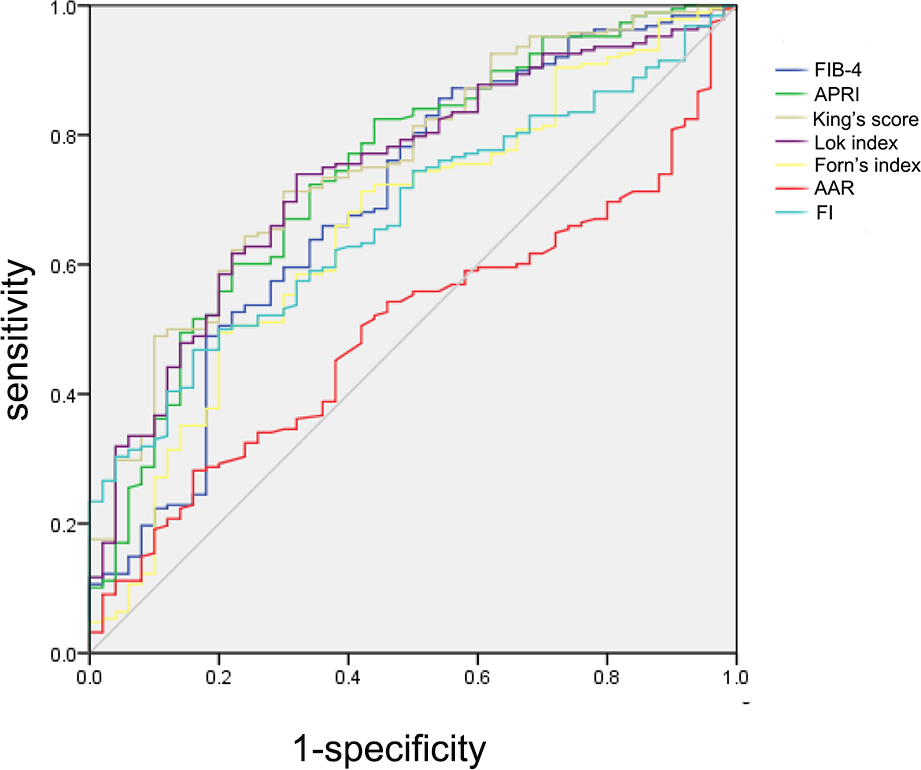
Performance of serum liver fibrosis indexes in the detection of CSPH. ROC curves showing the diagnostic accuracy of FIB-4, APRI, King’s score, Lok index, Forns index, AAR and FI in predicting the presence of CSPH in liver cirrhosis patients.

To further assess the diagnostic performance of recently proposed noninvasive fibrosis indexes in detecting CSPH, ROC curves were plotted (Fig 1). The optimized cutoffs for each noninvasive fibrosis index were calculated from the AUC analysis, as shown in Table 2. The diagnostic performance of these fibrosis indexes in the detection of CSPH is also shown. King’s score, APRI and the Lok index exhibited the best performance, as indicated by AUCs of 0.755, 0.742 and 0.740, respectively. APRI also exhibited the highest accuracy (76.89%) and the lowest -LR (0.31). Overall, the AUC of King’s score was significantly higher than that of FIB-4 (p = 0.0002), Forns index (p = 0.0002) and AAR (p < 0.0001). Overall, all of the fibrosis indexes exhibited good performance, with all PPVs > 85%; however, none could rule out CSPH with adequate sensitivity and reliability due to a low NPV (< 60%).

**Table 2:** Performance of serum liver fibrosis in the prediction of CSPH.

	FIB-4	APRI	King’s score	Lok index	Forns index	AAR	FI
**Cutoff**	2.72	0.73	23.47	1.30	11.05	1.08	-24.82
**Sensitivity (%)**	85.64	82.45	71.28	73.94	49.47	28.72	46.81
**Specificity (%)**	46.00	56.00	70.00	68.00	80.00	88.00	84.00
**PPV (%)**	85.64	87.57	89.93	89.68	90.29	90.00	91.67
**NPV (%)**	46	45.91	39.33	40.97	29.63	24.72	20.58
**Accuracy (%)**	76.89	76.89	71.01	72.69	55.46	58.82	57.56
**+LR**	1.59	1.87	2.38	2.31	2.47	2.39	2.93
**-LR**	0.31	0.31	0.41	0.38	0.63	0.81	0.63
**AUC(95% CI)**	0.694(0.631–0.751)	0.742(0.682–0.797)	0.755(0.695–0.808)	0.740(0.680–0.795)	0.657(0.593–0.717)	0.500(0.435–0.566)	0.694(0.631–0.751)

Abbreviations: CSPH, clinical significant portal hypertension; APRI, AST-to-platelet ratio index; AAR, AST-to-ALT ratio; FI, fibrosis index; PPV, positive predictive value; NPV, negative predictive value; LR, likelihood ratio; AUC, area under the curve; and CI, confidence interval.

### Performance of serum noninvasive liver fibrosis indexes in the detection of SPH

**Fig 2:**
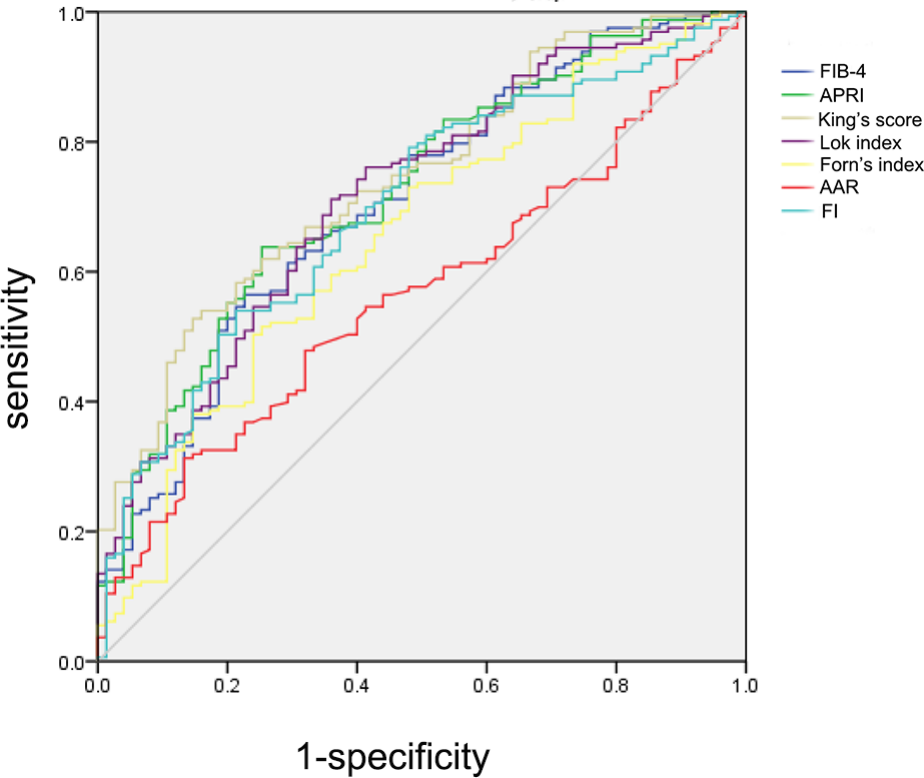
Performance of serum liver fibrosis indexes in the detection of SPH. ROC curves showing the diagnostic accuracy of FIB-4, APRI, King’s score, Lok index, Forns index, AAR and FI in predicting the presence of SPH in liver cirrhosis patients.

An ROC analysis was also used to assess the diagnostic performance of each noninvasive test in the detection of SPH (Fig 2). The optimized cutoff value and diagnostic performance of each serum liver fibrosis index in the detection of SPH are shown in Table 3. King’s score, APRI and the Lok index showed the best performance, as indicated by AUCs of 0.742, 0.722 and 0.717, respectively. The Lok index exhibited the highest accuracy (68.91%) with the lowest -LR (0.45). King’s score exhibited a high accuracy of 67.23% with the highest +LR (3.60). Moreover, the AUC of King’s score was significantly higher than that of FIB-4 (p = 0.0341), Forns index (p = 0.0008) and AAR (p = 0.0006). Taken together, none of the investigated fibrosis indexes, except King’s score, which had a PPV > 88.66%, were able to detect or rule out SPH with adequate sensitivity and reliability due to a low PPV (< 85%) and a low NPV (< 60%).

**Table 3:** Performance of serum liver fibrosis indexes in predicting SPH.

	FIB-4	APRI	King’s score	Lok index	Forns index	AAR	FI
**Cutoff**	4.77	1.10	35.17	1.40	11.09	1.59	-25.36
**Sensitivity (%)**	56.44	63.8	52.76	71.17	50.31	31.29	53.99
**Specificity (%)**	77.33	74.67	85.33	64	76	86.67	78.67
**PPV (%)**	84.40	84.56	88.66	81.12	82	83.61	84.62
**NPV (%)**	44.96	48.69	48.39	50.53	41.3	36.72	44.03
**Accuracy (%)**	63.03	67.23	67.23	68.91	58.4	48.74	61.34
**+LR**	2.49	2.52	3.60	1.98	2.10	2.35	2.53
**-LR**	0.56	0.48	0.55	0.45	0.65	0.79	0.58
**AUC(95% CI)**	0.706(0.643–0.763)	0.722(0.661–0.778)	0.742(0.681–0.796)	0.717(0.655–0.773)	0.652(0.588–0.713)	0.567(0.501–0.630)	0.698(0.635–0.755)

Abbreviations: SPH, severe portal hypertension; APRI, AST-to-platelet ratio index; AAR, AST-to-ALT ratio; FI, fibrosis index; PPV, positive predictive value; NPV, negative predictive value; LR, likelihood ratio; AUC, area under the curve; and CI, confidence interval.

### Combination of noninvasive liver fibrosis indexes for the prediction of HVPG

To increase the performance of the liver fibrosis indexes, combinations of two of the three fibrosis indexes that showed the best performance (King’s score, APRI and the Lok index), with an AUC > 0.7, were evaluated. The combination of King’s score (cutoff 23.47) and the Lok index (cutoff 1.30) was tested since this combination predicted the presence of CSPH with the highest PPV (95.38%) and +LR (5.49). The overall performance of the model was as follows: 52.7% sensitivity, 90.4% specificity, 95.38% PPV, 33.7% NPV, 57.56% accuracy, 5.49 +LR and 0.52 -LR. Among 238 patients, King’s score and the Lok index agreed in 137 (57.56%) cases. In 119 (50%) cases, King’s score was ≥ 23.47, and the Lok index was ≥ 1.30. For predicting the presence of SPH, the combination of King’s score (cutoff 35.17) and the Lok index (cutoff 1.40) exhibited the best performance with the highest PPV (93.48%) and +LR (7.11). The overall performance of the model was as follows: 34.55% sensitivity, 94.7% specificity, 93.48% PPV, 40.99% NPV, 42.72% accuracy, 7.11 +LR and 0.66 -LR. Among 238 patients, King’s score and the Lok index agreed in 106 (44.53%) cases. In 68 (28.57%) cases, King’s score was ≥ 35.17, and the Lok index was ≥ 1.40.

By applying this model combining King’s score (cutoff 23.47) and the Lok index (cutoff 1.30), more than half of the patients with a high risk of CSPH were identified for further examination of HVPG.

### Performance of serum liver fibrosis indexes in different subgroups

A subgroup analysis was conducted to investigate the influence of different clinical parameters on the performance of the fibrosis indexes in the prediction of PH. Seven single noninvasive fibrosis indexes and combinations of indexes with the best diagnostic performance were assessed by a subgroup analysis, including different Child-Pugh classes (Child-Pugh A and Child-Pugh B/C), different cirrhosis etiologies (viral cause and non-viral cause)and different gender(male and female).

No differences were observed in the AUC of any single fibrosis index among the different Child-Pugh classes, different etiology groups and different gender groups(Table 4, S1 Table). In addition, in the different etiology groups, the fibrosis indexes generally performed better in the prediction of CSPH and SPH in the viral-cause group than in the non-viral-cause group; however, the difference was not statistically significant, as shown in Table 4.

**Table 4:** Performance of serum liver fibrosis indexes among different subgroups in the prediction of CSPH and SPH.

**Prediction of CSPH (AUC 95%CI)**				
	**Patients with Child-Pugh A**	**Patients with Child-Pugh B/C**	**Patients withviral cause**	**Patients withnon-viral cause**
**FIB-4**	0.696(0.601–0.780)	0.607(0.517–0.692)	0.744(0.662–0.815)	0.674(0.575–0.763)
**APRI**	0.738(0.645–0.817)	0.711(0.624–0.788)	0.759(0.677–0.828)	0.709(0.611–0.794)
**King’s score**	0.744(0.652–0.823)	0.721(0.635–0.797)	0.784(0.705–0.850)	0.717(0.620–0.802)
**Lok index**	0.678(0.582–0.764)	0.687(0.599–0.766)	0.744(0.662–0.815)	0.711(0.613–0.796)
**Forns index**	0.680(0.585–0.766)	0.566(0.475–0.653)	0.716(0.632–0.790)	0.648(0.548–0.740)
**AAR**	0.511(0.414–0.607)	0.579(0.489–0.666)	0.545(0.457–0.631)	0.561(0.459–0.658)
**FI**	0.521(0.423–0.617)	0.694(0.606–0.772)	0.689(0.604–0.766)	0.658(0.558–0.749)
**Prediction of SPH (AUC 95%)**				
	**Patients with Child-Pugh A**	**Patients with Child-Pugh B/C**	**Patients withviral cause**	**Patients withnon-viral cause**
**FIB-4**	0.720(0.626–0.801)	0.640(0.551–0.723)	0.744(0.662–0.815)	0.672(0.572–0.761)
**APRI**	0.708(0.645–0.817)	0.723(0.637–0.799)	0.740(0.662–0.815)	0.682(0.583–0.771)
**King’s score**	0.724(0.631–0.805)	0.740(0.655–0.814)	0.768(0.687–0.836)	0.701(0.603–0.788)
**Lok index**	0.686(0.590–0.771)	0.625(0.535–0.709)	0.713(0.629–0.788)	0.701(0.602–0.787)
**Forns index**	0.694(0.599–0.779)	0.580(0.490–0.667)	0.690(0.605–0.767)	0.638(0.538–0.731)
**AAR**	0.559(0.461–0.653)	0.536(0.446–0.625)	0.564(0.476–0.649)	0.596(0.495–0.692)
**FI**	0.599(0.501–0.691)	0.598(0.507–0.683)	0.705(0.621–0.780)	0.694(0.596–0.781)

Abbreviations: CSPH, clinically significant portal hypertension; SPH, severe portal hypertension; AUC, area under the curve; CI, confidence interval; APRI, AST-to-platelet ratio index; AAR, AST-to-ALT ratio; and FI, fibrosis index.

For the prediction of CSPH and SPH using both King’s score and the Lok index, the data were as follows: among 135 viral-cause patients, King’s score and the Lok index agreed in 86 (63.70%) and 66 (48.89%) cases, and 78 (57.78%) and 52 (38.52%) cases were screened out, respectively. Among 103 non-viral-cause patients, King’s score and the Lok index agreed in 62 (61.16%) and 44 (42.72%) cases, and 51 (49.51%) and 21 (20.39%) cases were screened out, respectively. Therefore, the noninvasive screening model may be more applicable to patients with cirrhosis of viral etiology.

## Discussion

PH is a common complication of cirrhosis and contributes to the development of a series of complications. Studies have indicated that HVPG measurements perform well in the assessment of fibrosis or cirrhosis regardless of etiology and in the prediction of liver-related variceal hemorrhage[26,27]. In addition, recent guidelines indicate that HVPG measurements also provide prognostic information, such that changes in HVPG are associated with a relevant consequent outcome[28]. However, due to the invasiveness, requirement for advanced technical expertise and high costs associated with HVPG measurements, the introduction of simple, noninvasive screening and diagnostic methods would represent a major clinical advancement. Therefore, we intend to develop noninvasive methods as a first-line screening tool for the identification of patients at risk for PH whom may benefit from HVPG measurement.

In patients with cirrhosis, PH is associated with both increased IVR and increased portal inflow[29,30]. Liver fibrosis results in vascular compression due to collagen deposition around the sinusoids and the formation of regenerative nodules, which play major roles in the increase in IVR[31]. Recently, other studies have considered several noninvasive fibrosis indexes for the prediction of HVPG. In a prospective study, Vipin Verma reported that an APRI score ≥ 1.09 exhibited acceptable accuracy for the prediction of SPH, with an AUC of 0.716 (95% CI 0.574–0.858), 66% sensitivity, 73% specificity, 85% PPV, 47% NPV and 68% accuracy; however, that study included only one fibrosis index[32]. In another study, Eun Ju Cho et al investigated the diagnostic value of noninvasive markers, such as APRI, Forns index, FIB-4, Lok index and liver stiffness (LS) as determined by FibroScan, in predicting PH in patients with alcoholic cirrhosis[33]. The results of that study indicated that LS most accurately predicted CSPH in patients with compensated alcoholic cirrhosis. However, the study was limited to the field of alcoholic cirrhosis. In another study, acoustic radiation force impulse imaging (ARFI), transient elastography (TE) and APRI exhibited high diagnostic accuracy for CSPH in only 88 patients[34]. Currently, the incorporation of noninvasive methods, including serum-based markers and sonography-based methods, is increasingly used for the assessment of liver fibrosis or cirrhosis [35–38]; these methods provide a novel means of PH assessment. In Wei Zhang’s study, the combination of Fib-4 and FibroScan achieved a maximum AUC of 0.833 and accuracy of 77.8 for PH prediction [39]. However, the combination of these two methods remains insufficient for the assessment of PH. To date, no study has evaluated the diagnostic efficacy of the most recently proposed serum liver fibrosis indexes in predicting portal pressure in patients with cirrhosis.

For the abovementioned reason, we evaluated seven simple serum markers that were recently proposed for the noninvasive diagnosis of liver fibrosis; however, none of them showed sufficient accuracy or predictive values, with AUCs less than 0.80 for the diagnosis of CSPH and SPH. Additionally, we determined that King’s score, APRI and the Lok index exhibited better diagnostic accuracy than the other noninvasive indexes. However, their diagnostic accuracy was not significantly different from most comparable analytic parameters. Interestingly, all of the serum liver fibrosis indexes that were considered in the present study included at least one of the variables independently associated with PH grade, such as AST value, PLT count and ALB. The less-than-satisfactory results and their potential explanations are as follows: first, the noninvasive markers utilized in this study were first used for the assessment of liver fibrosis or liver cirrhosis in patients with chronic hepatitis C[17–23]; therefore, the different etiologies may have influenced the results. Second, liver fibrosis is not the only factor that may cause PH, as mentioned above; thus, the use of liver fibrosis indexes to predict HVPG may lead to slightly inaccurate results.

To further increase performance, combinations of the noninvasive fibrosis indexes were evaluated. It was previously suggested that combinations of several noninvasive methods might increase the diagnostic performance compared with that of single-index methods in the prediction of EV[8]. Based on recent recommendations, we aimed to predict PH, which is defined as CSPH, especially in patients with clinically relevant complications, such as EV and ascites; additionally, we aimed to predict SPH, which reflects the risk of variceal hemorrhage[40]. The results showed that the combination of King’s score (cutoff 23.47) and the Lok index (cutoff 1.30) predicted the presence of CSPH with the highest PPV and +LR. Since half of the patients had a King’s score greater than 23.47 and a Lok index greater than 1.30, a large number of patients would be selected by this method to undergo HVPG measurement to diagnose CSPH. For the prediction of SPH, the combination of King’s score (cutoff 35.17) and the Lok index (cutoff 1.40) also showed the highest PPV and +LR; however, only 20% of patients were identified with this method. Since CSPH precedes SPH, the model for screening CSPH may be used to establish an early diagnosis of PH for timely treatment. Combinations of single noninvasive indexes provide a tool for selecting patients with a high risk of CSPH for whom HVPG measurement may be more urgent, with a relatively small number of misdiagnosed cases. Such noninvasive first-line screening methods are especially needed, as HVPG measurement is not widely available, and rational allocation of resources may be critical in certain countries and regions.

The subgroup analysis of different cirrhosis etiologies revealed an interesting phenomenon: not only a single fibrosis index but also the combined screening model may be more applicable to patients with cirrhosis of viral etiology. This result was consistent with our previous univariate analysis, which showed that etiology was a variable associated with HVPG grade. We speculate that these results may be attributed to the fact that all of the evaluated noninvasive indexes were initially applied to patients with viral hepatitis. However, the present study included a limited number of cases; therefore, additional studies are required to confirm the positive preliminary results.

In conclusion, our study suggests that serum liver fibrosis indexes possess modest diagnostic accuracy in the detection of PH in patients with cirrhosis. However, the combination of King’s score (cutoff 23.47) and the Lok index (cutoff 1.30) may be used as an initial screening tool to identify cirrhosis patients who are at very high risk of CSPH and to determine the need for further evaluation with HVPG measurements.

## Supporting information

S1 Appendix All relevant data within this paper are available in Appendix. (XLSX)

S1 Table Performance of serum fibrosis indexes in different sex subgroups for prediction of CSPH and SPH. (DOCX)
