# Supplementary material for: Diagnostic efficacy of noninvasive liver fibrosis indexes in predicting portal hypertension in patients with cirrhosis
Source: PLoS One. 2017 Aug 18;12(8):e0182969. doi: 10.1371/journal.pone.0182969 (PMC5562323; doi:10.1371/journal.pone.0182969)
Supplement: S1 Table — (DOCX) [file pone.0182969.s002.docx]

| Prediction of CSPH | | | |
| --- | --- | --- | --- |
|  |  | **Male Patients** **AUC(95% CI)** | **Female Patients  AUC(95% CI)** |
| FIB-4 | 0.676(0.598 - 0.748) | | 0.723(0.609 - 0.819) |
| APRI | 0.758(0.684 - 0.822) | | 0.711(0.596 - 0.808) |
| King’s score | 0.756(0.682 - 0.820) | | 0.750(0.638 - 0.841) |
| Lok index | 0.770(0.698 - 0.833) | | 0.671(0.554 - 0.774) |
| Forns’s index | 0.643(0.564 - 0.717) | | 0.685(0.569 - 0.786) |
| AAR | 0.523(0.443 - 0.602) | | 0.576(0.458 - 0.688) |
| FI | 0.678(0.600 - 0.749) | | 0.653(0.536 - 0.758) |
| Prediction of SPH |  | |  |
|  | **Male Patients  AUC(95% CI)** | | **Female Patients  AUC(95% CI)** |
| FIB-4 | 0.688(0.610 - 0.758) | | 0.740(0.627 - 0.833) |
| APRI | 0.718(0.641 - 0.786) | | 0.742(0.630 - 0.835) |
| King’s score | 0.729(0.654 - 0.796) | | 0.772(0.663 - 0.860) |
| Lok index | 0.735(0.660 - 0.801) | | 0.677(0.561 - 0.779) |
| Forns’s index | 0.630(0.550 - 0.705) | | 0.704(0.589 - 0.803) |
| AAR | 0.592(0.512 - 0.669) | | 0.545(0.427 - 0.659) |
| FI | 0.710(0.633 - 0.779) | | 0.665(0.549 - 0.769) |

**S1 Table. Performance of serum fibrosis indexes in different sex subgroups**

**for prediction of CSPH and SPH**
